# Retrospective investigation of >400 patients undergoing thoracic endovascular aortic repair with or without cerebrospinal fluid drainage

**DOI:** 10.1093/icvts/ivad178

**Published:** 2023-11-14

**Authors:** Charlotte Mutter, Julia Benk, Tim Berger, Stoyan Kondov, Salome Chikvatia, Frank Humburger, Martin Rösslein, Felix Ulbrich, Martin Czerny, Bartosz Rylski, Maximilian Kreibich

**Affiliations:** Department of Cardiovascular Surgery, University Heart Centre Freiburg, University Medical Centre Freiburg, Freiburg, Germany; Faculty of Medicine, Albert-Ludwigs-University of Freiburg, Freiburg, Germany; Department of Cardiovascular Surgery, University Heart Centre Freiburg, University Medical Centre Freiburg, Freiburg, Germany; Faculty of Medicine, Albert-Ludwigs-University of Freiburg, Freiburg, Germany; Department of Cardiovascular Surgery, University Heart Centre Freiburg, University Medical Centre Freiburg, Freiburg, Germany; Faculty of Medicine, Albert-Ludwigs-University of Freiburg, Freiburg, Germany; Department of Cardiovascular Surgery, University Heart Centre Freiburg, University Medical Centre Freiburg, Freiburg, Germany; Faculty of Medicine, Albert-Ludwigs-University of Freiburg, Freiburg, Germany; Department of Cardiovascular Surgery, University Heart Centre Freiburg, University Medical Centre Freiburg, Freiburg, Germany; Faculty of Medicine, Albert-Ludwigs-University of Freiburg, Freiburg, Germany; Faculty of Medicine, Albert-Ludwigs-University of Freiburg, Freiburg, Germany; Department of Anesthesiology and Intensive Care Medicine, University Medical Centre Freiburg, Freiburg, Germany; Faculty of Medicine, Albert-Ludwigs-University of Freiburg, Freiburg, Germany; Department of Anesthesiology and Intensive Care Medicine, University Medical Centre Freiburg, Freiburg, Germany; Faculty of Medicine, Albert-Ludwigs-University of Freiburg, Freiburg, Germany; Department of Anesthesiology and Intensive Care Medicine, University Medical Centre Freiburg, Freiburg, Germany; Department of Cardiovascular Surgery, University Heart Centre Freiburg, University Medical Centre Freiburg, Freiburg, Germany; Faculty of Medicine, Albert-Ludwigs-University of Freiburg, Freiburg, Germany; Department of Cardiovascular Surgery, University Heart Centre Freiburg, University Medical Centre Freiburg, Freiburg, Germany; Faculty of Medicine, Albert-Ludwigs-University of Freiburg, Freiburg, Germany; Department of Cardiovascular Surgery, University Heart Centre Freiburg, University Medical Centre Freiburg, Freiburg, Germany; Faculty of Medicine, Albert-Ludwigs-University of Freiburg, Freiburg, Germany

**Keywords:** Aortic dissection, Aortic aneurysm, Cerebrospinal fluid drainage, Thoracic endovascular aortic repair

## Abstract

**OBJECTIVES:**

The aim of this study was to analyse the risks and benefits of cerebrospinal fluid drainage (CSFD) placement in patients undergoing thoracic endovascular aortic repair.

**METHODS:**

Between 2009 and 2020, 411 patients underwent thoracic endovascular aortic repair in 1 institution where 236 patients (57%) received a preoperative CSFD. Patient and outcome characteristics were retrospectively analysed and compared between patients with and without preoperative CSFD placement.

**RESULTS:**

Preoperative CSFD was performed significantly more frequently in elective patients, especially those undergoing distal stent graft extension following frozen elephant trunk-stent placement (*P* < 0.001). Significantly fewer CSFD was placed in patients with acute aortic injury (*P* < 0.001). The incidence of permanent spinal cord ischaemia (SCI) was higher in patients without preoperative CSFD [10 patients (2%) vs 1 patient (0.2%), *P* = 0.001]. Postoperative CSFD was placed in 3 patients (0.7%). Severe CSFD-associated complications affected 2 patients (0.5%) namely, a subdural spinal haematoma causing permanent paraplegia in one of those 2 patients.

**CONCLUSIONS:**

CSFS placement is associated with low procedural risk and can potentially help to prevent SCI. However, the SCI incidence is most likely also associated with other preoperative factors including the patient’s haemodynamics. Hence, a general recommendation for placing a preoperative CSFD cannot be made when relying on the present evidence.

**Figure:**
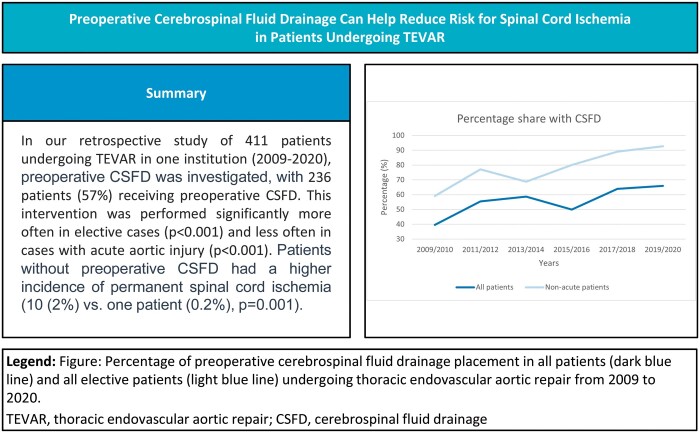

## INTRODUCTION

Thoracic endovascular aortic repair (TEVAR) has become the method of choice for treating pathologies of the descending aorta due to its significantly lower perioperative risk compared with open surgery [1]. However, periprocedural spinal cord ischaemia (SCI) remains a major problem with TEVAR; 2–10% of the patients are thus affected because of impaired spinal cord perfusion [2–4]. This serious complication can trigger devastating consequences for those affected such as permanent paraparesis and paraplegia [5].

A cerebrospinal fluid drainage (CSFD) can help surgeons assess spinal cord perfusion during TEVAR through intrathecal pressure monitoring. In the event of an increase in pressure, cerebrospinal fluid can be drained to lower the pressure and thus enable better blood flow to the structures at risk. The well-established therapeutic value of CSFDs as a therapeutic approach after the onset of SCI symptoms is enhanced by the option of its preoperative placement for prophylactic reasons. However, CSFD’s preoperative placement is controversial as a routine preventive measure in all patients [6–9]: to date, the benefit of CSFD’s routine protective and prophylactic nature has not been proven. Therefore, the latest guidelines do not recommend the general implantation of a preoperative CSFD in all patients. Its prophylactic placement should only be considered in patients identified as carrying a high risk of SCI [10–12].

Guidelines and extensive meta-analyses have identified a general lack of solid data for a higher class of recommendation regarding CSFD use in the treatment of thoracic aortic pathologies [10, 13]. The aim of this study was therefore to analyse the risks and benefits of preoperative CSFD placement in patients undergoing TEVAR.

## PATIENTS AND METHODS

### Ethics statement and study design

Our institutional review committee (University Hospital Freiburg, Germany) approved this retrospective study (IRB number: 20-1302), and the need for informed consent was waived due to the study’s retrospective nature. This is a retrospective single-centre study adhering to the ‘Interdisciplinary Cardio Vascular and Thoracic Surgery (ICVTS)’ author guidelines. All patients undergoing TEVAR in our institution between January 2009 and December 2020 were included in this study (*n* = 411).

### Patients

TEVAR was the treatment of choice for patients with descending aortic pathologies in case suitable landing zones and appropriate access vessels were present. All patients were included with acute and elective pathologies, which had the indication for TEVAR treatment, following the current guidelines [11]. Patients with indications for open repair or not suitable for any treatment were excluded. Computed tomography angiography scans were performed preoperatively, before discharge, during every follow-up visit and whenever clinically warranted. Our routine follow-up protocol includes visits to our dedicated aortic clinic after 6 months, 12 months and yearly thereafter.

### Endovascular approach

Our current endovascular approach has been described earlier [14, 15]. In short, we routinely access the vessel percutaneously to enable access to the common femoral artery using pre-closure techniques (Proglide, Abbott Medical, Chicago, IL, USA) while surgical cut down was used in the early study period. In case of a zone 1 TEVAR, supra-aortic double transposition is performed. In case of zone 2 TEVAR, we now routinely perform subclavian-to-carotid transposition or bypass. In clinically urgent scenarios, TEVAR is prioritized and left subclavian artery revascularization is done afterwards. In the early study period, routine subclavian revascularization was not performed in all zone 2 TEVARs. Today, we also routinely record and analyse intraoperative motor- and somatosensory-evoked potentials during all elective TEVAR interventions [16].

CSFD placement indications changed during the study period. In the early period, CSFD was placed on an individual case-by-case decision adhering to several clinical practice guidelines [10–12]. Today, a CSFD is routinely placed in all elective TEVAR procedures in the absence of contraindications (blood-clotting restrictions due to current medication or the pending intervention’s great urgency) on the day before surgery. Adhering to our standard operating procedure, CSFD placement is carried out by the departments of anaesthesia or neurosurgery routinely on the day before TEVAR. Coagulation is monitored beforehand, and oral anticoagulation is paused (platelet aggregation inhibitors 7 days prior, new oral anticoagulants 4 days prior and vitamin K antagonists are paused to attain a normal international normalized ratio). CSFD puncture is usually done at the L3/4 or L4/5 level. The catheter is secured by a suture, the puncture site is then covered with sterile foil dressing and the drainage tube is guided to the patient's flank using adhesive tape. Patients are then monitored for signs of complications such as loss of sensation, headache or bleeding at the injection site. Following TEVAR, patients are routinely monitored for 24 h in our intensive care unit, which entails invasive blood pressure measurements and continuous cerebrospinal fluid pressure monitoring. We usually place the drain drip circa 13–15 cm above the distal thoracic spine. The pressure of the liquor is continuously measured and displayed on the monitor. We do not let the liquor drip passively. If necessary, we detract about 5 ml of liquor and never >10 ml/h. Indications for the drainage of liquor are neurologic symptoms of the patients, an isolated decrease of the cerebrospinal fluid pressure of >5 mmHg or a cerebrospinal fluid pressure above 20 mmHg. Usually, the drains are removed the next day and patients are transferred to a normal ward.

### Data collection and definition of parameters

Data were collected retrospectively relying on our centre’s prospectively maintained aortic database. Patient characteristics, indications for TEVAR, perioperative details and clinical outcomes were analysed. The modified Rankin scale (mRS) was used to classify the severity of a postoperative stroke [17]. We distinguished between non-disabling (mRS 0–2) and disabling (mRS 3–6) strokes. SCI was specifically evaluated via clinical examination during the routine postoperative surveillance. Whenever SCI symptoms occurred, a CSFD was subsequently applied for therapeutic reasons in patients without a preoperative CSFD. We diagnosed a temporary SCI in case paraparesis or paraplegia was completely reversible, while any SCI causing persistent symptoms was labelled as permanent. Drainage-associated complications were classified as mild (headache, disconnection or loss of drainage or loss of cerebrospinal fluid), moderate (any bleeding complication) and severe (subdural and/or epidural haematoma).

### Statistical analysis

Data are presented as absolute and relative frequency or as median [first quartile, third quartile] and are compared among patients with and without preoperative CSFD placement. The Student’s *t*-test or Mann–Whitney *U*-test was applied to compare continuous variables when appropriate. Categorical variables were compared using the Chi-squared test. In case of small group sizes (*n* < 5), Fisher’s exact test was used. A value of *P* < 0.05 was considered statistically significant. Statistical analysis was conducted using IBM SPSS Statistic 21.0 (IBM-SPSS Inc., Armonk, NY, USA).

## RESULTS

### Patient characteristics

All patient characteristics are summarized in Table 1. Most patients were male and the cardiovascular risk profiles were similar between the groups with and without CSFD. Moreover, age did not differ statistically between the 2 cohorts. However, significantly more patients with preoperative CSFD suffered from coronary artery disease (*P* = 0.013).

**Table 1:** Patient characteristics

	All	Preoperative CSFD	No preoperative CSFD	*P*-value
***n***	**411**	**236 (57)**	**175 (43)**	
Age (years)	68 [59, 75]	68 [60, 75]	66 [57, 76]	0.200
Weight (kg)	80 [69, 90]	80 [69, 90]	80 [70, 90]	0.651
Height (m)	1.74 [1.67, 1.80]	1.74 [1.67, 1.80]	1.73 [1.65, 1.80]	0.206
BSA (m^2^)	1.96 [1.80, 2.10]	1.97 [1.80, 2.09]	1.96 [1.80, 2.12]	0.767
BMI (kg/m^2^)	26 [24, 29]	26 [23, 29]	27 [24, 29]	0.149
Male	264 (64)	151 (64)	113 (65)	0.917
Diabetic	44 (11)	19 (8)	25 (14)	0.053
Hyperlipidaemia	101 (25)	66 (28)	35 (20)	0.065
Hypertension	301 (73)	180 (76)	121 (69)	0.115
History of stroke	26 (6)	14 (6)	12 (7)	0.838
Chronic renal impairment	67 (16)	34 (14)	33 (19)	0.280
Dialysis	7 (2)	3 (1)	4 (2)	0.465
Chronic obstructive pulmonary disease	39 (9)	23 (10)	16 (9)	0.867
History of coronary artery disease	111 (27)	75 (32)	36 (21)	0.013
History of connective tissue disease	22 (5)	17 (7)	5 (3)	0.074

Values are represented as *n* (%) or median [first quartile, third quartile].

BMI: body mass index; BSA: body surface area; CSFD: cerebrospinal fluid drainage.

### Indication for thoracic endovascular aortic repair

The distribution of aortic dissections and aneurysms was similar between the groups, while CSFD placement was significantly more common in patients undergoing distal stent graft extension following the frozen elephant trunk procedure (*P* < 0.001) and significantly less common in patients treated for traumatic aortic rupture (*P* < 0.001). Correspondingly, a CSFD was significantly more common in non-acute (i.e. elective) cases and significantly less common in acute (i.e. emergent) cases (*P* < 0.001). Indications are summarized in Table 2.

**Table 2:** Indications for thoracic endovascular aortic repair

	All	Preoperative CSFD	No preoperative CSFD	*P*-value
***n***	**411**	**236 (57)**	**175 (43)**	
Type B dissection	112 (27)	60 (25)	52 (30)	0.371
Type non-A–non-B dissection	7 (2)	5 (2)	2 (1)	0.704
Type A dissection	11 (3)	7 (3)	4 (2)	0.765
Penetrating aortic ulcer	50 (12)	25 (11)	25 (14)	0.257
Aneurysm	110 (27)	72 (31)	38 (22)	0.055
Traumatic aortic injury	32 (8)	4 (2)	28 (16)	<0.001
Distal stent graft extension after frozen elephant trunk	67 (16)	56 (24)	11 (6)	<0.001
Other pathology	22 (5)	7 (3)	15 (9)	0.015
Acute event	194 (47)	54 (23)	140 (80)	<0.001
Non-acute event	217 (53)	182 (77)	35 (20)	<0.001

Values are represented as *n* (%).

CSFD: cerebrospinal fluid drainage.

### Periprocedural details

All patients with zone 1 TEVAR underwent a supra-aortic double transposition, while 10 patients underwent zone 2 TEVAR without carotid-to-subclavian bypass or transposition. Zone 3 was the most common proximal landing zone in all patients and significantly more common in patients without a preoperative CSFD (*P* < 0.001). Also, the frozen elephant stent graft portion was used as a proximal landing zone mostly in patients with a CSFD (*P* < 0.001). The vertebral and internal iliac arteries were patent in all patients. All periprocedural details are summarized in Table 3.

**Table 3:** Intraoperative data

	All	Preoperative CSFD	No preoperative CSFD	*P*-value
***n***	**411**	**236 (57)**	**175 (43)**	
Double transposition	9 (2)	6 (3)	3 (2)	0.739
Subclavian–carotid bypass	101 (25)	66 (28)	35 (20)	0.065
Subclavian–carotid transposition	2 (1)	2 (1)	0 (0)	0.510
Number of stent 1	222 (54)	121 (51)	101 (58)	0.230
Number of stent 2	147 (36)	88 (37)	59 (34)	0.468
Number of stent 3	36 (9)	24 (10)	12 (7)	0.291
Number of stent 4	4 (1)	3 (1)	1 (1)	0.640
Number of stents unknown	2 (1)	0 (0)	2 (1)	0.181
Patent left iliac artery	411 (100)	236 (100)	175 (100)	–
Patent right iliac artery	411 (100)	236 (100)	175 (100)	–
Patent left vertebral artery	408 (100)^a^	234 (100)	174 (100)	–
Patent right vertebral artery	408 (100)^a^	234 (100)^a^	173 (99)^a^	0.426
Proximal landing zone 1	9 (2)	6 (3)	3 (2)	0.739
Proximal landing zone 2	113 (27)	71 (30)	42 (24)	0.219
Proximal landing zone 3	162 (39)	72 (31)	90 (51)	<0.001
Proximal landing zone 4	66 (16)	36 (15)	30 (17)	0.684
Proximal landing zone after frozen elephant trunk	67 (16)	56 (24)	11 (6)	<0.001
X-ray time (s)	570 [366, 856]	560 [366, 850]	595 [361, 869]	0.861
Procedure time (min)	98 [68, 151]	99 [67, 154]	94 [68, 137]	0.888

Values are represented as *n* (%) or median [first quartile, third quartile] except where otherwise noted.

a No imaging available in 3 cases (*n* = 408).

CSFD: cerebrospinal fluid drainage.

### Outcome data

Patients without a preoperative CSFD presented with a significantly higher incidence of post-TEVAR acute kidney injury (*P* < 0.001), longer ICU stay (*P* < 0.001) and in-hospital mortality (*P* < 0.001). Outcome characteristics are summarized in Table 4.

**Table 4:** Outcome data

	All	Preoperative CSFD	No preoperative CSFD	*P*-value
***n***	**411**	**236 (57)**	**175 (43)**	
Spinal cord ischaemia temporary	12 (3)	5 (2)	8 (5)	0.253
Spinal cord ischaemia permanent	11 (3)	1 (0.4)	10 (6)	0.001
Non-disabling stroke	1 (0.2)	0 (0)	1 (1)	1.000
Disabling stroke	7 (2)	1 (0.4)	6 (3)	0.045
Acute kidney failure	20 (5)	3 (1)	17 (10)	< 0.001
Days on ICU	2 [1, 4]	2 [1, 3]	3 [1, 8]	< 0.001
Days in hospital	8 [6, 13]	8 [7, 12]	9 [4, 15]	0.982
In-hospital mortality	22 (5)	3 (1)	19 (11)	< 0.001

Values are represented as *n* (%) or median [first quartile, third quartile].

CSFD: cerebrospinal fluid drainage; ICU: intensive care unit.

A total of 11 patients (2.7%) suffered from a permanent SCI, which was significantly more frequent (*P* = 0.001) in patients without a preoperative CSFD. Of those 11 patients, 10 patients had not received a preoperative CSFD, and their TEVAR was done in an acute setting event (acute aortic dissection, acute aortic aneurysm). One patient underwent zone 1 TEVAR and double transposition for a chronic aortic aneurysm. Here, a CSFD had been inserted preoperatively but was found to be dislocated after surgery. The patient developed SCI and a second CSFD had to be reinserted. Unfortunately, his symptoms did not regress. Three of the 11 SCI patients expired in the hospital. All 12 of the observed cases of temporary SCI were evenly distributed between the 2 groups. Those patients’ symptoms regressed completely and had fully disappeared by the time of discharge without further intervention.

A postoperative drain was placed in 3 patients to exploit its therapeutic benefit for SCI after TEVAR, which proved beneficial in 2 patients. The other patient’s spinal ischaemia had been diagnosed prior to TEVAR and did not change after CSFD placement.

### Cerebrospinal fluid drainage details

CSFD details are summarized in Table 5. A total of 236 CSFDs were analysed regarding various aspects. Fluid was drained in 36% of patients and the CSFDs were removed after 23 h postoperatively on average. CSFD complications occurred in 38 patients (16%). However, complications were minor in 34 patients (14%). Two patients (0.8%) developed a severe CSFD complication, namely a subdural spinal haematoma causing permanent paraparesis of the legs in 1 patient and mild paresthesia of the legs in the other patient.

**Table 5:** Cerebrospinal fluid drainage details

	*n* = 236
Spinal fluid drained	84 (36)
Intraoperative drain (ml)	10 [5, 15]
Drained at ICU in (ml)	10 [5, 20]
Postoperative duration of CSFD (h)	23 [19, 26]
Medication during placement of CSFD	
Aspirin	8 (3)
Clopidogrel	0
NOAC	0
Phenprocoumon	0
LMWH	30 (13)
HMWH	8 (3)
Laboratory values before placement of CSFD	
INR	1.04 [0.99, 1.09]
PTT	31 [28, 34]
Platelet count	214 [172, 265]
Complications associated with CSFD	
Complications overall	38 (16)
Mild^a^	34 (14)
Headache of any kind	29 (12)
Disconnection or accidental loss	7 (3)
Loss of spinal fluid	7 (3)
Moderate	0
Bleeding at puncture site	0
Severe	2 (0.8)
Epidural haematoma	0
Subdural spinal haematoma	2 (0.8)

Values are represented as *n* (%) or median [first quartile, third quartile].

a If >1 complication occurred in the same patient, the case was counted only once.

CSFD: cerebrospinal fluid drainage; HMWH: high molecular weight heparin; ICU: intensive care unit; INR: international normalized ratio; LMWH: low molecular weight heparin; NOAC: new oral anticoagulants; PTT: partial thromboplastin time.

**Figure 1:**
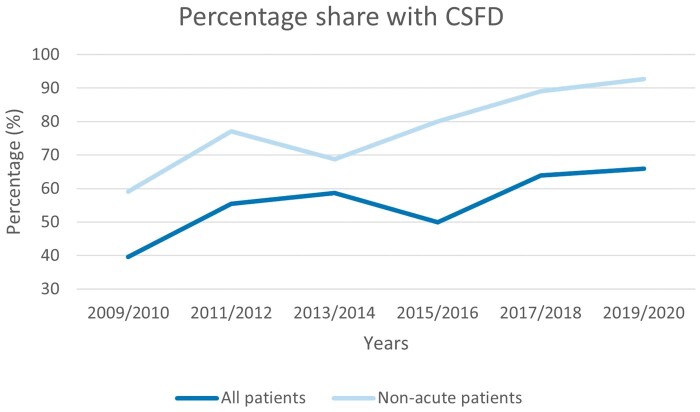
Percentage of preoperative cerebrospinal fluid drainage placement in all patients (lower/dark blue line) and all elective patients (upper/light blue line) undergoing thoracic endovascular aortic repair from 2009 to 2020.

There were 35 non-acute patients not receiving a preoperative CSFD in this study. In 2, the reason for not placing a CSFD was placement failure. Indications for not placing the drainage remain unclear in the remaining elective patients. We observed an increase in the use of a preoperative CSFD over time as depicted in Fig. 1. Today, almost all elective TEVAR patients receive a preoperative CSFD in our centre.

## DISCUSSION

This study’s most important findings are that: (i) CSFD placement is associated with low procedural risk, can be considered relatively safe and may help to prevent SCI; (ii) the SCI incidence is probably also associated with other preoperative factors including patients’ haemodynamics; hence (iii) a general recommendation placing a preoperative CSFD cannot be justified by the data we present here.

Our study cohort’s risk profile and medical history are in line with several other reports addressing the issue of TEVAR and reflect the overall high incidence of cardiovascular risk factors in patients with aortic diseases [18, 19]. The overall similarity of patients with and without a preoperative CSFD indicates that patient characteristics were not influencing the argument for or against CSFD placement in our study, but rather the treatment time period (Fig. 1) and the underlying pathology’s acuteness. Interestingly, we observed a significantly higher incidence of coronary artery disease in patients with a preoperative CSFD. This may be attributable to the significantly higher incidence of elective interventions, especially in patients undergoing a distal stent graft extension following frozen elephant trunk placement, as these patients generally undergo preoperative coronary artery angiography beforehand in our centre [20].

Indications for TEVAR differed significantly between groups; we noted a general trend favouring CSFD in the elective, planned interventional contexts and a higher incidence of emergent, acute interventions in patients without a preoperative CSFD. Today, it is our standard policy to place a preoperative CSFD before elective TEVAR to maximize spinal cord protection whenever possible. Through this approach, we have observed extremely low complication rates accompanying spinal cord perfusion, particularly in those patients undergoing distal stent graft extension [20]. Yet, in emergency situations, in patients with unstable haemodynamics or those still on preoperative oral anticoagulation therapy, immediate TEVAR is given priority over CSFD placement. We take other spinal cord protection approaches in these patients including elevating the arterial blood pressure, assuring an adequate haemoglobin level, fast-track concepts and serial postoperative neurological examination as previously reported [10, 14]. Nevertheless, these patients obviously still carry the highest risk for neurologic complications not only because of the lack of cerebrospinal fluid pressure monitoring but also because of spinal malperfusion related to their low cardiac output and catecholamine treatment [21, 22].

The periprocedural details here reflect our current revascularization approach regarding the left subclavian artery in 101 of the 113 zone 2 implantations. In stable patients, we follow current European consensus recommendations to maintain antegrade flow to the left arm, cerebellum and spinal cord [23]. Both a CSFD and subclavian revascularization in zone 2 implantations are the cornerstone of our spinal cord protection regime [24]. Interestingly, we observed a significantly higher incidence of zone 3 implantations in patients without a preoperative CSFD. Since fewer CSFDs were placed during our study’s early period and in acute scenarios, it could be that the implanting surgeon was hesitant to cover the left subclavian artery in non-elective scenarios and opted to use zone 3 as a quicker non-ideal landing zone. Also, the higher incidence of distal stent graft extensions following the frozen elephant trunk procedure in patients with a CSFD may well be attributable to the procedure’s generally elective nature.

Patients with a preoperative CSFD presented with a lower incidence of postoperative SCI, and the difference reached statistical significance in patients suffering from permanent SCI. However, the absence of a preoperative CSFD alone does not probably explain the higher incidence of spinal cord malperfusion, even though a preoperative CSFD may have helped in monitoring and preventing SCI and is beneficial as a postoperative therapeutic tool when SCI symptoms occur after surgery. In fact, patients without a preoperative CSFD also presented a significantly higher incidence of other relevant adverse outcomes including stroke, kidney failure and in-hospital mortality. The negative postoperative course was most likely influenced by preoperative characteristics in most of these patients, particularly the acuteness of the underlying pathology. Hence, it is plausible that patients presenting an acute pathology (possibly in haemodynamic shock) carry a higher risk for an overall negative outcome; moreover, these are the patients who will not likely receive a preoperative CSFD because of their TEVAR treatment’s urgency to stabilize the patient. Other risk factors for SCI include extended aortic coverage and Ishimaru’s distal landing zone 5–10 [4, 25, 26]. However, today, the determination of whether a patient carries a ‘high risk’ and therefore a CSFD should be considered (as official guidelines suggest) is not based on a widely accepted scoring system.

As mentioned above, the periprocedural events (i.e. acute kidney injury, longer stay on intensive care unit, higher in-hospital mortality) are mainly influenced by the complexity of the given patient’s pathology and condition, so that drawing a straight causal connection exclusively with the drainage issue is unlikely. The exact effects of applying drainage or not have not yet been proven by any study. Nevertheless, the positive therapeutic effect of drainage is well accepted if an SCI occurs. The rate of permanent SCI after TEVAR in this study compares very well to the described rate of 3.9% in the most recent meta-analysis by Gaudino *et al.* [2]. The fact that 10 of the 11 affected patients in this study underwent surgery within an extremely acute context, and therefore, no preoperative CSFD placement was possible, explains the significantly higher rate of SCI in the group without a CSFD. In the 1 remaining patient, who also suffered from SCI but had received a CSFD preoperatively, it became apparent after the procedure that the drain had malfunctioned due to incorrect positioning. In other words, we observed no incident of permanent SCI while CSFD was functioning.

The therapeutic utility of postoperatively inserted CSFD was revealed in 3 patients. The drainage did not alleviate SCI symptoms in only 1 patient; however, this proved to be a pre-existing SCI caused by the aortic pathology itself, and thus, any post-TEVAR improvement was unlikely. Our observations lead us to assume that drainage can both protect against SCI development (since permanent SCI occurred exclusively in individuals without CSFD) and also provide a benefit as a therapeutic tool for new-onset SCI. Note, however, that this assessment is based solely on the observations made in this study that do not allow any final conclusions to be drawn because of the relatively low number of events. Nevertheless, the evidence in our data is in line with the findings of the largest randomized trial to date on the use of CSFD [27].

Lastly, this study investigated the details and complications of the CSFD itself. Following our clinical routine, the postoperative drainage duration was 23 h, reflecting our routine postoperative monitoring period in the intensive care unit, where the CSFD is removed before the transfer to the normal ward. However, as others have reported, the comparison of drainage-associated complications with data from other publications is complicated by inconsistencies in how they are classified. Most studies make a distinction between mild, moderate and severe complications, whereby allocation to the first 2 categories is especially inconsistent [2, 13, 28, 29]. Our classification follows that applied in published papers on the topic, to enable maximum comparability. Severe complications in the form of 2 observed cases of subdural haematoma at a rate of 0.8% were below the 1.8% average frequency of occurrence ranging to 4.6% in current meta-analyses [2, 28]. Thanks to the low complication rates and transient nature of most CSFD-associated complications, the use of CSFD for the prevention and therapy of SCI can be considered safe overall. It is therefore not surprising that, as a result of such positive experience with the CSFD in our department, we tend to apply it routinely. Our aim is to take a preoperative approach whenever possible to maximize spinal cord protection and to standardize procedures among all involved healthcare professionals when it comes to a CSFD. Nevertheless, particularly in the early study period, CSFD placement was infrequent in our department and the indication was usually determined by the individual surgeon’s decision. This highlights one of the major problems in assessing CSFD as a prophylactic tool. The objectification of the benefit can only be analysed retrospectively as long as the individual surgeon's decision as a soft factor exerts such a strong influence on the procedure. Since the benefit of identifying patients at increased risk is supported by several criteria in the current guidelines, but the decision for or against the device is never made solely in their basis, we need a carefully defined protocol within the department for a randomized trial. Such protocols will likely differ, even if only slightly, from those in other departments—a factor that further complicates any comparison of their results [10].

### Limitations and strengths

Our study is limited by its sample size and retrospective nature. Although our data reveal a strong advantage of using CSFD, as no cases of SCI were observed in patients with (functional) drainage, it is clearly limited by the low number of events. However, this investigation is nevertheless one of the largest case series analysing risks and benefits of preoperative CSFD in patients undergoing TEVAR, and we think it makes a substantial contribution to future evaluations. Note that there are significant differences regarding baseline parameters as discussed in the prior discussion section.

## CONCLUSIONS

CSFD placement is associated with low procedural risk, can be considered relatively safe and may help to prevent SCI. However, the incidence of SCI is most likely associated with other preoperative factors including the patient’s haemodynamic status. Therefore, our data do not justify making a general recommendation for the application of preoperative CSFD.

## Data Availability

Access to the data that support the findings of this study is available from the corresponding author, Maximilian Kreibich, upon reasonable request. Access is limited by privacy and ethical restrictions. **Charlotte Mutter:** Conceptualization; Data curation; Formal analysis; Investigation; Methodology; Project administration; Software; Visualization; Writing—original draft. **Julia Benk:** Conceptualization; Data curation; Formal analysis; Methodology; Project administration; Resources; Supervision; Validation; Visualization; Writing—original draft. **Tim Berger:** Conceptualization; Methodology; Project administration; Resources; Supervision; Validation; Writing—review & editing. **Stoyan Kondov:** Conceptualization; Methodology; Project administration; Resources; Supervision; Validation; Writing—review & editing. **Salome Chikvatia:** Conceptualization; Methodology; Project administration; Resources; Validation; Writing—review & editing. **Frank Humburger:** Conceptualization; Methodology; Project administration; Resources; Validation; Writing—review & editing. **Martin Rösslein:** Conceptualization; Methodology; Resources; Validation; Writing—review & editing. **Felix Ulbrich:** Conceptualization; Methodology; Resources; Validation; Writing—review & editing. **Martin Czerny:** Conceptualization; Methodology; Project administration; Resources; Supervision; Validation; Writing—review & editing. **Bartosz Rylski:** Conceptualization; Methodology; Project administration; Resources; Supervision; Validation; Writing—review & editing. **Maximilian Kreibich:** Conceptualization; Data curation; Formal analysis; Funding acquisition; Investigation; Methodology; Project administration; Resources; Software; Supervision; Validation; Writing—original draft. Interdisciplinary CardioVascular and Thoracic Surgery thanks Naomichi Uchida, Gabriele Piffaretti and the other, anonymous reviewer(s) for their contribution to the peer review process of this article.

## ABBREVIATIONS

CSFD: Cerebrospinal fluid drainage
mRS: Modified Rankin Scale
SCI: Spinal cord ischaemia
TEVAR: Thoracic endovascular aortic repair
